# Effect of treatment zone decentration on axial length growth after orthokeratology

**DOI:** 10.3389/fnins.2022.986364

**Published:** 2022-10-20

**Authors:** Shuxian Zhang, Hui Zhang, Lihua Li, Xiaoyan Yang, Shumao Li, Xuan Li

**Affiliations:** ^1^Clinical College of Ophthalmology, Tianjin Medical University, Tianjin, China; ^2^Tianjin Key Lab of Ophthalmology and Visual Science, Tianjin Eye Hospital, Tianjin, China; ^3^Tianjin Eye Hospital Optometric Center, Tianjin, China; ^4^Nankai University Affiliated Eye Hospital, Nankai University, Tianjin, China

**Keywords:** orthokeratology, myopia, axial length, decentration, refraction

## Abstract

**Objective:**

To study the effect of treatment zone (TZ) decentration on axial length growth (ALG) in adolescents after wearing the orthokeratology lenses (OK lenses).

**Materials and methods:**

This retrospective clinical study selected 251 adolescents who were fitted OK lenses at the Clinical College of Ophthalmology, Tianjin Medical University (Tianjin, China) from January 2018–December 2018 and wore them continuously for >12 months. The age of the subjects was 8–15 years, spherical equivalent (SE): −1.00 to −5.00 diopter (D), and astigmatism ≤ 1.50 D. The corneal topography were recorded at baseline and 1-, 6-, and 12-month visits, and the axial length (AL) were recorded at baseline and 6-, 12-month visits. The data of the right eye were collected for statistical analysis.

**Results:**

The subjects were divided into three groups according to the decentration distance of the TZ after wearing lenses for 1 month: 56 cases in the mild (<0.5 mm), 110 in the moderate (0.5–1.0 mm), and 85 in the severe decentration group (>1.0 mm). A significant difference was detected in the ALG between the three groups after wearing lenses for 6 and 12 months (*F* = 10.223, *P* < 0.001; *F* = 13.380, *P* < 0.001, respectively). Among these, the 6- and 12-month ALG of the mild decentration group was significantly higher than that of the other two groups. Multivariable linear regression analysis showed that age, baseline SE, and 1-month decentration distance associated with the 12-month ALG (*P* < 0.001, *P* < 0.001, and *P* = 0.001, respectively).

**Conclusion:**

The decentration of the TZ of the OK lens affected the growth of the AL in adolescents, i.e., the greater the decentration, the slower the ALG.

## Introduction

Currently, myopia accounts for about 30% of the global population and is expected to rise to 50% by 2050 (Holden et al., 2016). Similarly, the current situation of myopia is not optimistic in China. The overall myopia rate of children and adolescents is about 50%, and that in high school students is 80%, among which about 10–20% is high myopia (Chen M. et al., 2018; Dong et al., 2020). The condition is irreversible and incurable once it occurs, and high myopia causes various complications, such as cataract, glaucoma, retinal detachment, and macular degeneration (Flitcroft, 2012; Tsai et al., 2019). In the population with high myopia, the risk of fundus lesions is >40 times higher than emmetropia (Hu et al., 2020). These complications are closely related to the excessive axial length growth (ALG) in high myopia. The goal of myopia control is to slow the progression and prevent a low myopia from becoming a moderate or high myopia (Bullimore and Brennan, 2019).

Orthokeratology lenses (OK lenses) are rigid gas-permeable contact lenses (RGPCL) with a special reverse geometric design. These lenses can reshape the front surface of the cornea during sleep, temporarily reduce myopia, and improve visual acuity (Lian et al., 2014). Recent studies (Cho et al., 2005; Kakita et al., 2011) have confirmed that compared to single-vision spectacle, OK lenses can reduce the ALG by 40–60%. However, some OK lens wearers experienced that the center of the OK lens treatment zone (TZ) is often inconsistent with the center of the pupil. Presently, only a few studies have assessed the influence of TZ decentration on ALG with a small sample size (Wang and Yang, 2019; Chen et al., 2020).

The present study aimed to observe the effect of different decentration states of the lenses on the ALG after wearing OK lenses and explore the association between the ALG and individual factors, such as age, gender, spherical equivalent (SE), astigmatism, corneal curvature, corneal astigmatism, pupil diameter, TZ decentration distance, and TZ decentration direction.

## Materials and methods

### Subjects

This retrospective clinical study was conducted to collect adolescent myopia subjects who underwent OK lens fitting at the Clinical College of Ophthalmology, Tianjin Medical University (Tianjin, China), from January 2018–December 2018. The inclusion criteria were as follows: (1) Age: 8–15 years; (2) Subjective refraction under cycloplegia: −5.00 diopter (D) ≤ SE ≤ −1.00 D, astigmatism ≤ 1.50 D; (3) The flat K (FK) of the cornea was 39.00D ∼ 46.00D; (4) Monocular corrected visual acuity of OK lenses ≥ 0.8 (Visual acuity expressed as decimal visual acuity); (5) Both eyes were fitted with OK lenses for the first time and worn continuously for ≥12 months. Exclusion criteria: (1) Stop wearing the lenses for ≥7 days for any reason during the follow-up, including corneal and conjunctival adverse events, lens broken, and lens parameter adjustment, etc; (2) Obvious keratoconjunctival complications, glare, diplopia, or any other symptoms during the follow-up; (3) Contraindications to wearing contact lenses for ocular and systemic diseases. This study adhered to the tenets of the Declaration of Helsinki and was approved by the Institutional Ethical Committee Review Board of Tianjin Eye Hospital (Scientific Research Review No. 2022005). The right eye data were collected for statistical analysis to avoid the symbiosis of binocular myopia.

### Fitting of the orthokeratology lenses

All subjects were fitted by a team of one ophthalmologist and three optometrists. The OK lenses were fitted according to the manufacturer’s instructions. The OK lenses selected in this study had a four-zone reverse-geometric design (Euclid Systems Orthokeratology; Euclid System, Herndon, VA, USA) manufactured at the Boston Equalens II material, with a nominal DK 90 × 10^–11^ (cm^2^/s) (mlO_2_/mL ⋅ kPa^–1^), and the lenses diameters were 10.0–11.2 mm. The difference of the total diameter of the lens was located in the fitting arc. The diameter of the optical zone fixed at 6.2 mm; the width of the reverse arc was 0.5 mm; the width of the fitting arc ranged from 0.9 to 1.5 mm, and the curvature range of the fitting arc ranged from 39.00D to 46.00D; the width of the peripheral arc was 0.5 mm, the curvature radius of the peripheral arc was 11.5 mm.

### Refraction and corneal curvature examination

Cycloplegic refraction was performed at baseline. Three drops of 0.5% Topicamide/0.5% deoxyepinephrine hydrochloride were dropped at 5-min intervals. Subjective refraction was assessed at least 30 min after the last drop of eye drops. SE = spherical diopter + 1/2 cylindrical diopter. The FK and steep K (SK) of the cornea were measured by an auto refraction keratometer (ARK-510A, Nidek, Aichi, Japan).

### Measurement of axial length

The AL was measured at baseline and at 6- and 12-month follow-up using an ocular biometric (IOL-Master 500, Carl Zeiss, Ag, Jena, Germany). Five consecutive measurements were recorded, and the average value was taken for data analysis at each follow-up. The difference between the AL and the baseline value at each follow-up was considered the ALG.

### Corneal topography and evaluation of treatment zone decentration

**FIGURE 1:**
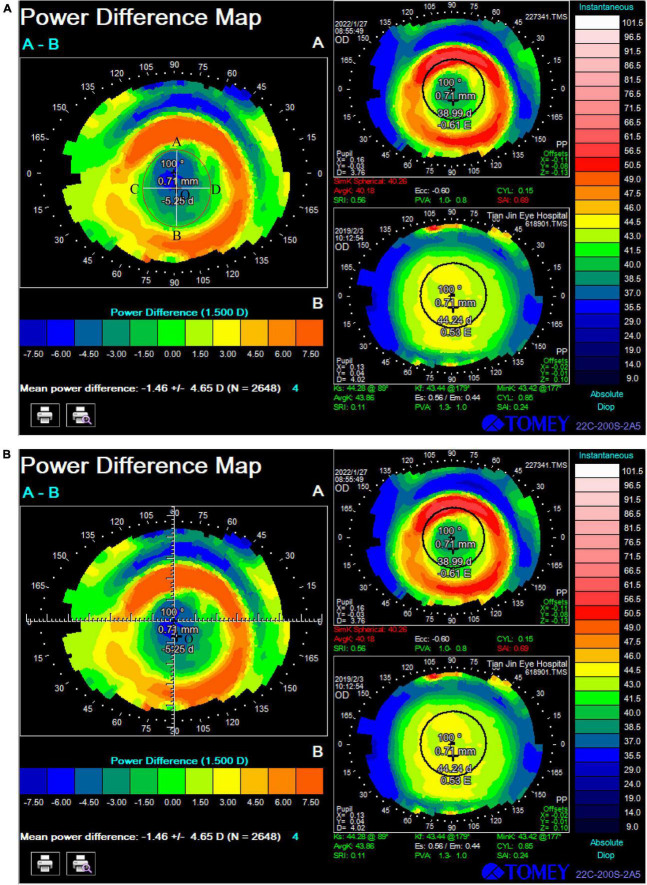
Calculation method of treatment zone decentration: **(A)** Decentration distance: the area surrounded by the red circle is the treatment zone; line segments AB and CD were the horizontal and vertical diameters of the treatment zone; O point was the central point of the treatment zone. The distance from O point to the center of the pupil is 0.71 mm. **(B)** The position of the central point of the treatment zone (O point) on the coordinate axis: the pupil center was taken as the origin, and the horizontal (*X*)-axis and vertical (*Y*)-axis were set up. The decentration angle of O point was displayed by the topographic map software, which was 100°, The treatment zone decentration along *X*-axis and *Y*-axis was calculated as follows: X = cos 100 × 0.71, Y = –sin 100 × 0.71.

Corneal topography (TMS-4, Tomey, Nagoya, Japan) was measured after four consecutive measurements of each eye; the best imaging quality and good gaze were selected for data analysis. All measurements were conducted by the same technician. The decentration distance and direction were calculated according to the method of Guo et al. (2022): (1) decentration distance: the TZ was defined as the area enclosed by points with 0 diopter changes on the tangential subtractive topography maps, and the tolerance of each zero point at the edge of the TZ was ± 0.10 D. Briefly, the refractive power change was obtained by hovering the cursor above each reference point and the measurement shown on the image was recorded. As shown in Figure 1A, the area surrounded by the red circle is the TZ, line segments AB and CD were the horizontal and vertical diameters of the TZ, respectively. They intersect at O point, which was the central point of the TZ. When the cursor is hovered over the O point and the topographic map software, it automatically displays the distance between the O point and the pupil center, i.e., the decentration distance. This decentration distance was divided into mild decentration (<0.5 mm), medium decentration (0.5–1 mm), and severe decentration (>1.0 mm) according to the method of Tsai and Lin (2000). (2) The position of the central point of the TZ on the coordinate axis was calculated as follows: the pupil center was taken as the origin, and the horizontal (*X*)-axis and vertical (*Y*)-axis were set up, as shown in Figure 1B. The decentration angle of the central point of the TZ (O point) was displayed by the topographic map software (α-angle), i.e., the included angle between the connecting line between the center of the TZ and the pupil center and the *X*-axis. The TZ decentration along *X*-axis and *Y*-axis was calculated as follows: X = cos α × decentration distance, Y = sinα × decentration distance, with positive signs representing nasal or superior decentration.

### Pupil diameter

Pupil diameter were determined with Corneal topography (TMS-4, Tomey, Nagoya, Japan). In the Tomey topography system, pupil diameter value was provided for each map. Corneal topographic maps were measured in a windowless examination room with lighting of approximate 300–310 lx. In this study, pupil diameters were recorded from baseline topographic maps of subjects for statistical analysis.

### Measurements and follow-up visits

After dispensing the lens, the subjects were instructed to wear and care for them properly. Moreover, the subjects were advised to wear their OK lenses every night for 8–10 h. The examinations were performed before any lens wear (baseline) and 1 day, 1 week, 1 month, and 3 months, and then every 3 months until 1 year. Each follow-up included naked visual acuity, subjective and objective refraction (ARK-1, Nidek, Aichi, Japan), slit-lamp anterior segment examination (SL-D701, Topcon, Yamagata, Japan), and corneal topography. The AL was measured at 6- and 12-month follow-ups. Corneal topography data at 1-, 6-, and 12-month visits and AL at 6- and 12-month visits were used for statistical analysis.

### Statistical analysis

SPSS statistical software (version 20.0, IBM SPSS, Chicago, IL, USA) was used for data analysis. Quantitative data were tested for normality using quantile-quantile plots, described as mean ± standard deviation, and the classification data were described as the number of cases and percentages. Repeated measures analysis of variance (ANOVA) was used to compare the decentration distance at different follow-ups after wearing OK lenses. For consistent comparison between baseline parameters of different groups, one-way ANOVA was used for continuous variables, and the chi-square test was used for classification variables. The 6- and 12-month ALG between different groups was compared by one-way ANOVA. Pearson’s correlation analysis was used to assess the simple association between the 12-month ALG and decentration distance achieved at 1-month visit, and Spearman’s correlation analysis was used to assess the association between the 12-month ALG and decentration direction. This association was further examined using linear regression analysis. Variables, including baseline age, gender, SE, astigmatism, FK, SK, corneal astigmatism, pupil diameter, and 1-month decentration distance and direction were first examined using univariate linear regression analysis. Variables with statistically significant associations (*P* < 0.05) with the 12-month ALG in univariate analyses were entered into the multivariate regression model to further test whether it has statistical significance on the 12-month ALG. *P* < 0.05 was considered a statistically significant difference.

## Results

### Baseline data

A total of 251 subjects (251 eyes) were enrolled in this study, including 112 (44.6%) males and 139 (55.4%) females. The mean baseline age was 10.47 ± 1.97 years, the mean SE was −3.23 ± 1.14 D, and the mean astigmatism was −0.55 ± 0.58 D. FK and SK were 42.82 ± 1.29 D and 44.03 ± 1.40 D, respectively. The mean corneal astigmatism was 1.21 ± 0.56 D, and the mean baseline AL was 24.94 ± 0.83 mm. The mean baseline pupil diameter was 4.14 ± 0.30 mm.

### Decentration distance at different follow-up visits

The decentration distance at 1-, 6-, and 12-month visits was 0.83 ± 0.32 mm, 0.84 ± 0.33 mm, and 0.84 ± 0.32 mm, respectively. The difference was not statistically significant (*F* = 2.877, *P* = 0.066), indicating stable decentration of the lenses at the 1-month visit. The subsequent studies were statistically analyzed based on the data of the 1-month visit.

### Decentration distribution

**FIGURE 2:**
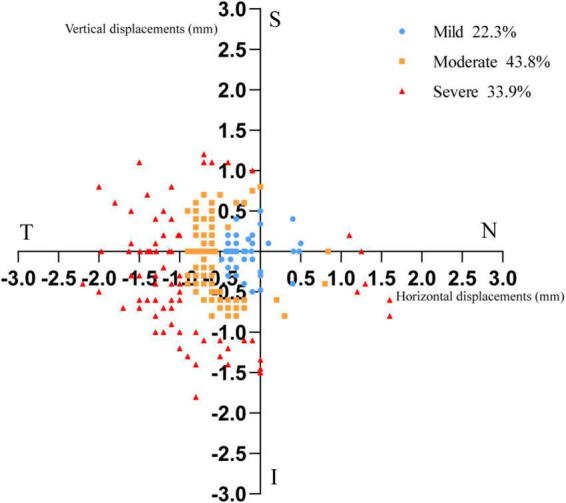
The distribution of the center of the treatment zone for all subjects. Each point depicted in the figure represents the location of the center point of each subject’s treatment zone in an axis with the pupil center as the origin. Each plotted point shows the distance and angle of the center of treatment zone from the pupil center. The spacing of each grid on the coordinate axis represents 0.5 mm on the cornea. (T: temple; N: nasal; S: superior; I: inferior).

After wearing the lens for 1 month, the decentration direction of the TZ was as follows: superior 2 cases (0.8%), superonasal 4 cases (1.6%), nasal 5 cases (2.0%), inferonasal 9 cases (3.6%), inferior 9 cases (3.6%), inferotemporal 129 cases (51.4%), temporal 36 cases (14.3%), and superotemporal 57 cases (22.7%). Among these, inferotemporal decentration was the most commonly observed phenotype. Figure 2 is the distribution of the center of the treatment zone for all subjects.

### Baseline data in different groups

At 1-month visit, 56 (22.3%) cases in the mild decentration group (30 males and 26 females), with a mean decentration of 0.41 ± 0.09 mm; 110 (43.8%) cases in the moderate decentration group (51 males and 59 females), with a mean decentration of 0.76 ± 0.13 mm; and 85 (33.9%) cases in the severe decentration group (31 males and 54 females), with a mean decentration of 1.20 ± 0.14 mm were assimilated. No significant difference was detected in the gender between different groups (χ^2^ = 3.918, *P* = 0.141), while astigmatism, SK, and corneal astigmatism showed statistical significance among the three groups (*P* < 0.001, *P* = 0.030, and *P* < 0.001, respectively). The other baseline parameters did not show any significant difference among the three groups (*P* > 0.05), as shown in Table 1.

**TABLE 1:** Baseline data in different decentration groups (mean ± standard deviation).

Variables	Mild	Moderate	Severe	*F*-value	*P*-value
Age (years)	10.18 ± 2.03	10.48 ± 1.94	10.64 ± 1.97	0.938	0.393
SE (D)	−3.11 ± 1.08	−3.18 ± 1.18	3.31 ± 1.16	2.879	0.082
Astigmatism(D)	−0.33 ± 0.38	−0.51 ± 0.54	0.76 ± 0.66	10.750	<0.001
FK(D)	43.07 ± 1.47	42.63 ± 1.23	42.91 ± 1.22	2.500	0.084
SK (D)	44.03 ± 1.64	43.80 ± 1.30	44.34 ± 1.31	3.571	0.030
Corneal astigmatism (D)	0.96 ± 0.45	1.17 ± 0.51	1.43 ± 0.61	13.766	<0.001
AL(mm)	24.75 ± 0.83	24.91 ± 0.86	25.09 ± 0.77	2.860	0.059
Pupil diameter (mm)	4.10 ± 0.29	4.18 ± 0.29	4.12 ± 0.30	1.756	0.175

### Axial length growth

Statistically significant differences were observed in the 6- and 12-month ALG among the three groups, as shown in Table 2.

**TABLE 2:** Axial length growth (ALG) in different groups.

Variables	Mild	Moderate	Severe	*F*-value	*P*-value
6-month ALG (mm)	0.12 ± 0.14	0.05 ± 0.12	0.02 ± 0.15	10.223	<0.001
12-month ALG (mm)	0.26 ± 0.19	0.14 ± 0.17	0.09 ± 0.19	13.380	<0.001

**FIGURE 3:**
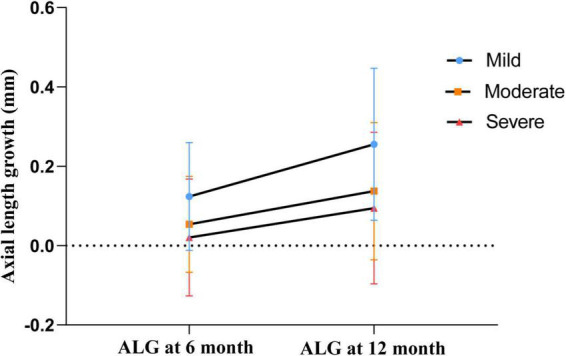
6- and 12-month axial length growth among different groups. The mean values are represented by blue dots (mild decentration group), orange squares (moderate decentration group) and red triangles (severe decentration group), respectively. Error bars represent standard deviation of the mean.

Moreover, the 6- and 12-month ALG of the mild decentration group was significantly higher than that of the moderate (*P* = 0.004 and *P* < 0.001) and the severe decentration groups (*P* < 0.001 and *P* < 0.001), while no significant difference was noted between the moderate and severe decentration groups (*P* = 0.194, *P* = 0.244), as shown in Figure 3.

### 12-month axial length growth and individual factors

**FIGURE 4:**
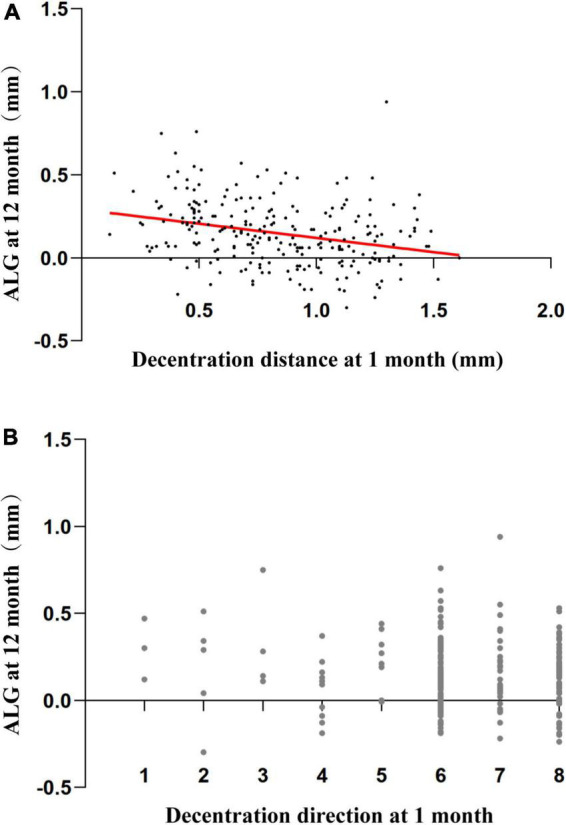
Association between 12-month axial length growth and decentration factors. **(A)** Scatterplots demonstrating the association between the 12-month axial length growth and 1-month decentration distance. There was a significant association between the parameters (Pearson’s correlation analysis: *r* = –0.289, *P* < 0.001) **(B)** Scatterplots demonstrating the association between the 12-month axial length growth and 1-month decentration direction (1: superior; 2: superonasal; 3: nasal; 4: inferonasal; 5: inferior; 6: inferotemporal; 7: temporal; 8: superotemporal). There was no association between 12-month axial length growth and 1-month decentration direction (Spearman’s correlation analysis: *r* = –0.028, *P* = 0.657).

The 12-month ALG showed a significantly simple negative association with the 1-month decentration distance (*r* = −0.289, *P* < 0.001), but showed no association with 1-month decentration direction (*r* = −0.028, *P* = 0.657), as shown in Figure 4.

Baseline age, SE, and 1-month decentration distance were significantly associated with 12-month ALG in univariate and multivariate linear regression analysis. Corneal astigmatism was associated with 12-month ALG in the univariate analysis, which became statistically insignificant in the multivariate regression model. Pupil diameter has no statistically associated with 12-month ALG in univariate or multivariate regression analyses, as shown in Table 3.

**TABLE 3:** Linear regression analyses of 12-month ALG and individual factors.

	Univariate model		Multivariate model	
	β-value (95% CI)	*P*-value	β-value (95% CI)	*P*-value
Gender (boys)	0.027(−0.021,0.075)	0.269	–	–
Age (years)	−0.041(−0.052,−0.030)	<0.001	−0.035 (−0.045, −0.024)	<0.001
SE(D)	0.051(0.033,0.070)	<0.001	0.034 (0.017, 0.052)	<0.001
Astigmatism (D)	0.028(−0.014,0.069)	0.190	—	—
FK (D)	0.016(0.006,0.037)	0.156	—	—
SK (D)	0.009(−0.008,0.026)	0.310	—	—
Corneal astigmatism (D)	−0.044(−0.087,−0.001)	0.043	−0.005 (−0.044, 0.034)	0.793
Pupil diameter (mm)	−0.012(−0.094,0.070)	0.771	–	–
1-month decentration distance (mm)	−0.171(−0.242,−0.100)	<0.001	−0.114 (−0.182, −0.046)	0.001
1-month decentration direction	−0.010(−0.027,0.007)	0.262	–	–

## Discussion

In the present study, the 6- and 12-month ALG of the mild decentration group was significantly higher than that of the moderate and severe decentration groups. Baseline age and 1-month decentration negatively associated with 12-month ALG, while baseline SE is positively associated with 12-month ALG in univariate and multivariate linear regression analysis. However, the decentration direction and pupil diameter did not affect ALG. Corneal astigmatism was only associated with 12-month ALG in univariate regression analysis but not significantly in multivariate regression analysis.

### The distribution of the treatment zone decentration

This study showed that the distribution of decentration direction is most common in the inferotemporal, followed by the superotemporal and the temporal, which is similar to the previous findings (Hiraoka et al., 2009; Chen J. et al., 2018). The elevated nasal and temporal sides of the human cornea are asymmetric. Typically, the nasal side is higher than the temporal side (Jaisankar et al., 2020). Hence, the lens is prone to deviate to the temporal side, which is relatively low in elevation. Chen et al. (2017) analyzed the corneal elevation data collected at the 8-mm chord and confirmed the inferotemporal quadrant to be the paracentral corneal region lowest in elevation. Therefore, our study showed the inferotemporal decentration was most common (as shown in Figure 2), that is consistent with previous findings.

### Treatment zone decentration and axial length growth

The exact mechanism by which OK lens delays the progression of myopia remains unclear. The OK lens induced relative corneal refractive power shift (RCRPS) to produce myopic defocusing on the peripheral retina, thereby delaying the growth of AL, i.e., the “peripheral defocusing hypothesis,” which has been recognized by several investigators (Charman et al., 2006; Kang and Swarbrick, 2016; Santodomingo-Rubido et al., 2017). Recent studies have shown that the spatial distribution of relative corneal refractive power (RCRP) is more significant than a simple sum in delaying AL progression. Yang et al. (2021) and Jiang et al. (2021), respectively confirmed that the closer the RCRP is to the center of the cornea and the faster RCRP reaches the peak, the slower the ALG. The TZ decentration increases the corneal asymmetry and shifts the RCRP on one side of the cornea toward the center (Wang et al., 2018, 2021). Lin et al. (2021, 2022) demonstrated that the TZ decentration was significantly positively associated with summed RCRP within 1–2 mm chord radius, and RCRP was negatively associated with ALG. Therefore, a high TZ decentration can delay AL progression.

Another opinion was that there was a threshold of RCRP. Only when the defocus exceeds this threshold will the “stop” signal start to delay the ALG (Kang and Swarbrick, 2016). For the same average RCRP, the greater the variation in all directions, the more uneven the distribution and the slower the ALG (Gu et al., 2019). The current study did not find any association between decentration direction and 12-month ALG. Thus, it could be deduced that the RCRP need not reach or be above the threshold in every direction but only for a specific direction to reach the threshold. The larger the decentration distance of the lens, the more uneven the corneal shape and the greater chance for RCRP to reach the threshold, thus delaying ALG.

In the process of corneal reshaping, the characteristic morphological changes of the cornea will also increase the higher-order aberrations (Lau et al., 2020b). This phenomenon suggests that mechanisms other than myopic defocus may be involved in the inhibitory effect of the OK lenses on ALG (Lau et al., 2020a). Hiraoka et al. (2015) showed that the aberrations also played a major role in ALG delay after wearing the OK lenses; among all aberration factors, coma has the closest association with ALG. Some studies have shown that the aberration changes caused by lens decentration were mainly increased coma and spherical aberration (Lau et al., 2020b). These findings indicated that asymmetric corneal shapes, rather than concentric and radially symmetric shapes, have a considerable effect on the retardation of AL. Lin et al. (2020) indicated that increased myopia defocuses in the temporal retina is caused by the decentration of lenses toward the temporal side, resulting in 3 diopters myopic defocus of the temporal retina, while astigmatism and higher-order aberration also increased. Therefore, we hypothesized that the combination of these changes slowed the ALG after the TZ decentration.

### Other factors and axial length growth

In the current study, greater baseline corneal astigmatism was related to slower axial growth in univariate regression analysis. This could be attributed to larger corneal astigmatism leading to lens decentration (Chen et al., 2017; Gu et al., 2019; Jiang et al., 2019). However, the association became statistically insignificant after adjusting for the effects of age, SE, and decentration distance using multivariate linear regression analysis. Therefore, the decentration of the TZ and not corneal astigmatism affected ALG.

The ALG of myopic children might be affected by several factors. Previous studies have shown that age is a baseline parameter significantly associated with axial growth, and the younger the age, the faster the axial growth (Zhong et al., 2015; Chen et al., 2020; Lin et al., 2021; Sun et al., 2022), which is consistent with the current findings. However, the association between ALG and baseline SE is also controversial (Vincent et al., 2021). Some studies reported a significant negative association between ALG and baseline SE, as the subjects had a wide range of baseline SE, usually between −0.75 D and −6.00 D (Wang et al., 2017; Lin et al., 2021; Yang et al., 2021). While in the studies that reported a lack of association between the two factors (Zhong et al., 2015; Hu et al., 2019), the subjects’ baseline SE was in a limited intermediate range between −1.00 D and −4.00 D. In this study, subjects had −1.00 D to −5.00 D range of baseline SE, and the sample size was large. The results showed a significant negative association between ALG and baseline SE.

Since the pupil diameter will affect the measurement of peripheral refraction (Mathur and Atchison, 2013; Romashchenko et al., 2020) and higher-order aberration (Applegate et al., 2007), the pupil diameter is a factor that must be considered when studying the influencing factors of ALG after wearing OK lenses. Our study showed that baseline pupil diameter do not affecting 12-month ALG. Two other longitudinal studies (Wang et al., 2017; Zhao et al., 2020), similar to our findings, found no association between pupil diameter and ALG in children Treated with OK lenses. Conversely, the study of Chen et al. (2012) showed that after 2 years’ follow-up, larger pupil diameter in dark environment was significantly associated with smaller ALG. In another comparative study of European children wearing OK lenses and single-vision spectacle (Santodomingo-Rubido et al., 2013), there was negative association between pupil diameter and ALG in children in the OK lens group. We found that studies showing an association between pupil diameter and ALG, measured pupil diameter in the dark. While the measurement of our baseline pupil diameter is based on the detection value of Tomey topographic software, which is measured in a bright environment. During the daytime, particularly near work tasks (Gislen et al., 2008), children’s pupil diameters are much smaller than that in dark environments. Therefore, studying the relationship between pupil diameter and ALG in bright environment is more instructive for clinical work.

### Treatment zone decentration and adverse events

Severe Ortho-K lens decentration increased the risk of corneal adverse events, such as staining and indentation in the corneal epithelium (Maseedupally et al., 2016). In addition, severe TZ decentration increased the high-order aberration (Chen J. et al., 2018), reduced the contrast sensitivity (Hiraoka et al., 2009), and caused visual interference, such as glare and glowering (Nti and Berntsen, 2020). Therefore, in clinical practice, increasing the TZ decentration is not encouraged.

### Limitations of this current study

Nevertheless, the present study has several limitations. Firstly, this is a retrospective study, and many factors, such as family history in refractive errors, duration of outdoor activities, and habits of reading, were not recorded and could potentially interfere with the findings. Secondly, according to the literature, we speculated that the decentration of the TZ increased the RCRP, but we did not measure the RCRP in different decentration groups, and hence the association between the decentration and the RCRP could not be tested. Thirdly, we also speculated that the decentration of the TZ increased the higher-order aberration; however, there was no measured aberration value. These speculations would be substantiated in future prospective studies with a rigorous design.

## Conclusion

In conclusion, the TZ decentration of OK lenses can affect the ALG in adolescent myopia patients, and the greater the decentration distance, the slower the ALG. Thus, delaying the ALG by artificially creating decentration is not advocated.

## Data availability statement

The original contributions presented in this study are included in the article/supplementary material, further inquiries can be directed to the corresponding author.

## Ethics statement

This study adhered to the tenets of the Declaration of Helsinki and was reviewed and approved by the Institutional Ethical Committee Review Board of Tianjin Eye Hospital (Scientific Research Review No. 2022005). This is a retrospective clinical study, and the data have been anonymized. The requirement for informed consent was therefore waived.

## Author contributions

SZ was responsible for data collection and analysis and manuscript drafting. XL contributed to the study design and revision of the manuscript. HZ and XY contributed to the data collection. LL provided the administrative, technical, and material support. SL conducted the statistical analysis. All authors made substantial intellectual contributions to this study and reviewed and approved the final version of the manuscript.
